# Evidence of the association between the Q2 mitochondrial group of *Bemisia tabaci* MED species (Hemiptera: Aleyrodidae) and low competitive displacement capability

**DOI:** 10.1371/journal.pone.0280002

**Published:** 2023-01-12

**Authors:** Bruno Rossitto De Marchi, Andre Bueno Gama, Hugh A. Smith

**Affiliations:** 1 Entomology and Nematology Department, Gulf Coast Research and Education Center, University of Florida, Wimauma, FL, United States of America; 2 Plant Pathology Department, Gulf Coast Research and Education Center, University of Florida, Wimauma, FL, United States of America; University of Saskatchewan College of Agriculture and Bioresources, CANADA

## Abstract

The whitefly, *Bemisia tabaci* (Gennadius), is one of the most serious agricultural pests worldwide. *Bemisia tabaci* is a cryptic species complex of more than 40 species among which the invasive MEAM1 and MED species are the most widespread and economically important. Both MEAM1 and MED present intraspecific genetic variability and some haplotypes are reported to be more invasive than others. MED can be further deconstructed into different genetic groups, including MED—Q1 and MED—Q2. However, distinct biological phenotypes discerning the different MED mitochondrial haplotypes are yet to be characterized. Competitive displacement and life-history trials were carried out between MED-Q2 and MEAM1 populations collected in Florida, USA. In addition, a phylogenetic analysis was carried out including populations from previous whitefly competitive displacement studies for identification and comparison of the MED mitochondrial groups. In contrast to other studies with MED—Q1, the MED–Q2 population from Florida is less likely to displace MEAM1 on pepper. In addition, both pepper and watermelon were a more favorable host to MEAM1 compared to MED–Q2 according to the life history trials.

## Introduction

Previous studies have described *Bemisia tabaci* as a complex of at least 40 cryptic species based on the mitochondrial cytochrome oxidase I (mtCOI) gene [1, 2]. *Bemisia tabaci* causes significant economic losses among agronomic, horticultural and ornamental crops through mechanical damage, transmission of viruses, and induction of plant disorders [3]. Among the species, the Middle East-Asia Minor 1 (also known as MEAM1, B biotype or *Bemisia argentifolii*) and the Mediterranean (MED or Q biotype) are the most damaging to agriculture. MEAM1 and MED presents intraspecific variability and only a few haplotypes are invasive and are globally distributed [4, 5]. In the United States, *B*. *tabaci* MEAM1 Gennadius (Hemiptera: Aleyrodidae) is the prevalent species found in agricultural crops throughout the country. MED was first reported in the United States in 2004 and has been reported from greenhouse-grown ornamental horticulture plants in 26 states [6]. In 2016, MED was confirmed to be established on ornamental plants in residential areas in various parts of south Florida [7], but has not established in field agriculture. MED is known to develop resistance rapidly to several groups of insecticides including the growth regulator pyriproxyfen [8], the neonicotinoids acetamiprid [8, 9], imidacloprid [10] and thiamethoxam [8], and the diamide cyantraniliprole [11].

The population dynamics of the *B*. *tabaci* species complex may be influenced by several factors including temperature, host plant, and insecticide use [8, 12, 13]. MED displaced other *B*. *tabaci* species in Mediterranean areas with intensive agriculture because of its ability to develop resistance to insecticides, tolerance to high temperatures and mild tolerance to humidity [8, 9, 13, 14]. In Brazil, there is evidence of MED being introduced at least twice [15, 16]. It is currently a major concern on bell pepper [*Capsicum annuum* Linnaeus (Solanaceae)] grown in greenhouses, a crop on which *B*. *tabaci* was a secondary pest before the MED introduction. MED was also found in Brazil in open fields in mixed infestations with MEAM1 on tomato [*Solanum lycopersicum* Linnaeus (Solanaceae)] and eggplant [*Solanum melongena* Linnaeus (Solanaceae)] [17]. In China, field surveys revealed a rapid displacement of MEAM1 and establishment of MED after its introduction in most of the locations assessed [18, 19]. The factors that caused the displacement in China are likely associated with the wide use of neonicotinoid insecticides, including acetamiprid, thiamethoxam, and nitenpyram, as standard treatments for many sucking pests, including whiteflies, on a variety of crops [18].

Recent studies revealed that the cryptic species status of *B*. *tabaci* MED [20, 21] may be more complex than previously thought. Phylogenetic analyses based on intraspecies genetic variability have placed MED species populations into four different genetic groups: Q1 and Q2, (originally present in western and eastern Mediterranean countries respectively), and Q3 and African silver-leafing (ASL), species originating in Africa [21, 22]. More recently, significant differences in host range among these genetic groups were reported revealing that Q1 is the most polyphagous and Q2 the least polyphagous [21]. In Uganda, *B*. *tabaci* MED-ASL was the most prevalent in a field survey and indicated to be among the most polyphagous whitefly species found, occurring on 33 out of 59 identified plant species [23]. These differences may have implications for *B*. *tabaci* MED management and invasiveness. There is a lack of information associating different MED haplotypes with competitive displacement capability, which may influence the pest status of newly-introduced MED in a given region or country. In earlier literature, including competition studies, the haplotype of MED was often not identified [24, 25]. Our hypothesis is that MED–Q2 is less likely than the more polyphagous MED–Q1 to outcompete MEAM1 in certain crops [21]. In this study, we collected a local MED–Q2 population and performed competition experiments to evaluate the ability of MED-Q2 to displace MEAM1 on pepper and watermelon [*Citrullus lanatus* Thunberg (Cucurbitaceae)]. These host plants were chosen because of their economic importance in Florida and other parts of the United States. Previous studies with these *B*. *tabaci* populations confirmed that MEAM1 will outcompete MED-Q2 on tomato by the third generation [26]. The proportion of males and females was recorded by the end of each breakdown in the competitive displacement trials. In addition, we evaluated the number of eggs laid and number of first instar nymphs of these populations on the same hosts. The mtCOI gene of the MED population used in this study was sequenced and included in a phylogenetic analysis along with MED field populations previously collected in the US and populations from other competition studies involving MED and MEAM1.

## Material and methods

### Whitefly colonies

Two colonies, one of *B*. *tabaci* MEAM1 and the other of *B*. *tabaci* MED-Q2, were reared in organdy covered cages on cotton plants [*Gossypium hirsutum* Linnaeus (Malvaceae)]. The MEAM1 colony was established in the 1990s from whiteflies collected near Bradenton, FL. The MED-Q2 colony (mtCOI GenBank accession OK086082) was established in July 2017 from whiteflies collected from hibiscus [*Hibiscus rosa-sinensis* Linnaeus (Malvaceae)] in Palm Beach County, FL. Each colony was maintained in a separate growth room at 27°C (±2°C), 50% to 75% RH and 14:10 (L:D) photoperiod.

### Competitive displacement studies

Trials were performed on two different host plants, pepper cv. Antebellum (Seminis, St. Louis, MO, USA) and watermelon cv. Mickylee (Seedway, Hall, NY, USA), grown in an insect-free growth room at the Gulf Coast Research and Education Center (GCREC), Wimauma, FL, under the same conditions used to maintain the whitefly colonies. The peppers were seeded in trays and transplanted to 15 cm pots 6 weeks after emergence. The watermelon seeds were planted directly into the pots used in the trials. Cages of different sizes were used to accommodate the growth of plants over time. The plants were placed in organdy cloth covered cages of 33x33x61cm (1^st^ generation breakdown), 45x45x129cm (2^nd^ generation breakdown) or 56x56x188cm (3^rd^ generation breakdown). Trays were placed underneath the pots containing plants, which were watered by filling the tray with water as needed. Trays were located outside the small cages and inside the medium and large cages, which in each case allowed for watering the plants without opening cages. Water was applied directly into the tray for small cages and into the tray through the cage mesh for medium and large cages.

For each plant host, twelve plants (4 plants for each of the 3 cage sizes/breakdown dates) were subjected to either of the three treatments: (1) 6 male and 6 female MEAM1, (2) 6 male and 6 female MED, (3) 6 males and 6 females of both MEAM1 and MED. Cages with these treatments were established for three different breakdown intervals: 30, 60 and 90 days after whitefly release for pepper and 30, 50 and 70 days after whitefly release for watermelon. Intervals were shortened for watermelon because powdery mildew tended to become established on plants in the growth rooms after about 60 days. Each treatment by breakdown date combination was replicated 4 times in a randomized complete block design requiring a total of 36 cages per host plant tested.

On the breakdown date, the cages were placed in a cold chamber at 4°C for at least 2 hours to immobilize whiteflies. Then, all the whiteflies were aspirated from each of the cages using a mechanical aspirator (model DOA-P704-AA; Fisher Scientific, Pittsburgh, Pennsylvania, USA). The collected whiteflies were then separated by gender, counted and transferred to vials containing 90% ethanol prior to molecular analysis for species identification.

### Whitefly life history trials

Five female and five male adult whiteflies were confined in clip cages attached to a leaf of either pepper or watermelon, resulting in a total of 10 leaves (10 replicates) per plant type. After 48 h, the clip cages and adults were removed. Daily observations of leaves were made and the following information was recorded: number of eggs, percentage egg hatch, and number of first instar nymphs. The generation time from egg to adult was also estimated. The number of nymphs reaching adulthood was recorded only for the pepper as powdery mildew infections interfered with multiple attempts to collect these data from watermelon.

### Data analysis

Data were analyzed in a general linear mixed model to quantify the effects of the independent variables ‘breakdown interval’, ‘whitefly species’, and ‘host plant’. The analysis was performed using the GLIMMIX procedure of SAS (Version 9.4, Cary, NC). We specify a Poisson distribution for count data (number of eggs, nymphs, and adults) and binomial distribution for proportion data (percentage of females, percentage hatch). Standardized residual and normality plots were used to determine the adequacy of model fit. Pairwise treatment differences for the fixed effects were obtained using the LSMEANS statement and LINES option (P = 0.05) using the post-hoc test Tukey.

### *Bemisia tabaci* cryptic species identification

Each cage resulted in a different total number of adult whiteflies recovered on the breakdown day. Some cages contained a few thousand whitefly adults. We performed molecular analysis on a random sample of 1000 individuals in cages containing more than that number of whiteflies. In cages containing equal or less than 1000 adult whiteflies, all the individuals were analyzed.

Total nucleic acid extraction was performed on individual whiteflies following a modified Chelex protocol [27]. Whitefly adults were homogenized in 40 μL of 5% Chelex solution in a 1.5 mL tube. The tube was vortexed for 10 seconds followed by incubation at 56°C for 15 min and at 99°C for 8 min. After centrifugation at 13,000 rpm for 5 min, the supernatant was collected and used as a template for the PCR amplification. The PCR to differentiate MEAM1 from MED was carried out using the primer pair Bem23F (5’-CGGAGCTTGCGCCTTAGTC-3’) and Bem23R (5’-CGGCTTTATCATAGCTCTCGT-3’), which amplifies a microsatellite locus of about 200 bp and 400 bp for MEAM1 and MED [28–30], respectively. The PCR product was visualized by electrophoresis in 2% agarose gel stained with Gel Red. Later, five individuals of each colony, MEAM1 and MED, were analyzed using PCR with the generic insect primers C1-J-2195 and TL2-N-3014, that amplify a fragment of the mtCOI [31] followed by PCR purification and Sanger sequencing of the fragment (GENEWIZ, South Plainfield, NJ, USA).

### Phylogenetic analysis

To improve understanding the genetic variability of *B*. *tabaci* in relation to previous competition studies, a phylogenetic analysis was performed. The dataset consisted of nucleotide sequences from this study as well as sequences retrieved from GenBank including previous whitefly competition studies [24, 25], previous whitefly surveys in the United States [6], and a global *B*. *tabaci* mtCOI dataset [32]. The dataset was submitted to a multiple sequence alignment using the MAFFT77 software within Geneious v9.1.5. Subsequently, a phylogenetic analysis was conducted using MrBayes v. 3.2.2 [33] running on the HiPerGator supercomputer (located at the University of Florida, Gainesville). Analyses were run for 30 million generations with sampling every 1000 generations. Each analysis consisted of four independent runs, each utilizing four coupled Markov chains. The run convergence was monitored by finding the plateau in the likelihood scores (standard deviation of split frequencies <0.0015). The first 25% of each run was discarded as burn-in for the estimation of a majority rule consensus topology and posterior probability for each node. Trees were visualized, edited and rooted using FigTree v1.4.2.

## Results

**Fig 1:**
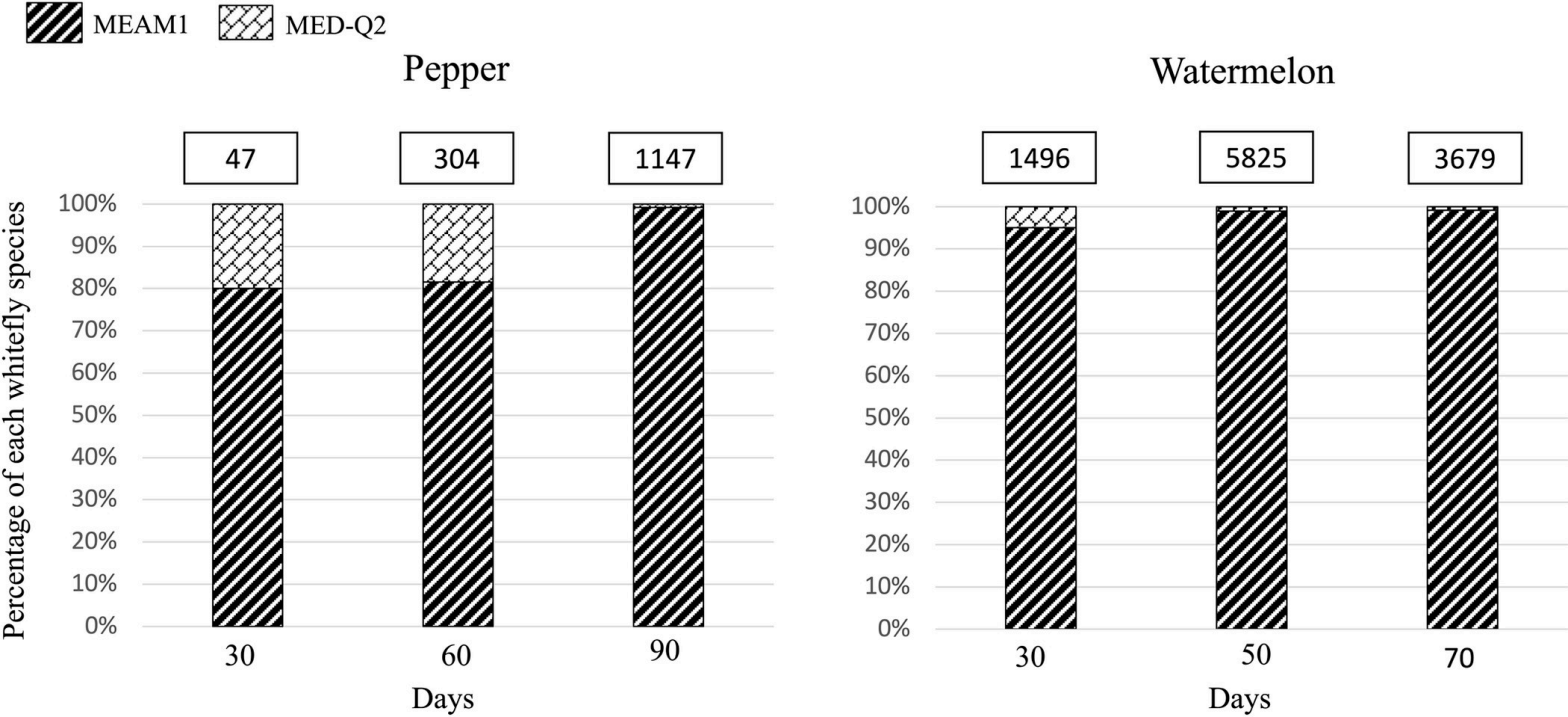
Percentage of each *Bemisia tabaci* species (MEAM1 or MED-Q2) collected at the first, second and third breakdowns on pepper or watermelon in the mixed treatment of the competitive displacement trial. Each cage was inoculated with six males and six females of each species. Numbers in the white boxes at the top of each column represent the total whitefly individuals (MEAM1 + MED-Q2) recovered in the four replicates by the end of the trial for each treatment.

The competitive displacement trials when whiteflies were mixed (MEAM1+MED-Q2) revealed that MEAM1 species was able to outcompete MED-Q2 species (>99% of the individuals sampled were identified as MEAM1) after 90 days on pepper and after 50 days on watermelon (Fig 1).

When whiteflies were inoculated alone (either MEAM1 or MED-Q2) in the watermelon trials, the mean number of MEAM1 adults recovered was higher than the mean number of MED-Q2 adults in the first (*F*_1,6_ = 2222.60; *P* < .0001), second (*F*_1,5_ = 9632.13; *P* < .0001) and third (*F*_1,6_ = 31867.5; *P* < .0001) breakdowns. In the pepper trials, the mean number of MEAM1 recovered was higher than MED-Q2 adults only in the second (*F*_1,6_ = 259.64; *P* < .0001) and third (*F*_1,6_ = 985.72; *P* = 0.0010) breakdowns (Table 1).

**Table 1:** Mean number of MEAM1 and MED-Q2 adults ± SE (males + females) from pepper and watermelon plants when inoculated with one species at 1^st^, 2^nd^ and 3^rd^ breakdown intervals.

Breakdown	Species	Pepper	Watermelon
1	MEAM1	11.5 ± 1.5 aB	1238.5 ± 116.2 aA
	MED-Q2	6.5 ± 3.4 aB	74.7 ± 10.4 bA
2	MEAM1	134.5 ± 58.4 aB	6823.7 ± 1976.4 aA
	MED-Q2	14.0 ± 6.6 bB	478.6 ± 140.9 bA
3	MEAM1	1556.0 ± 377.0 aB	17222.50 ± 3785.70 aA
	MED-Q2	35.00 ± 4.00 bB	2772.00 ± 1102.76 bA

Lowercase letters are used to compare means within the same column and breakdown group; uppercase letters are used to compare means in the same row. Means within the same cell (Breakdown × Species) followed by different lowercase letters are significantly different at *P* = 0.05; means within the same row followed by a different uppercase letter are significantly different at *P* = 0.05.

These trials also revealed that watermelon is a more suitable host than pepper for both MEAM1 and MED-Q2. The mean number of MEAM1 adults recovered from the watermelon was higher than from pepper in the first (*F*_1,3_ = 997.95; *P* < .0001), second (*F*_1,3_ = 8134.65; *P* < .0001) and third (*F*_1,1_ = 18901.8; *P* = 0.0046) breakdowns. In addition, more MED-Q2 adults were recovered from the watermelon than the pepper in the first (*F*_1,3_ = 142.68; *P* = 0.0013), second (*F*_1,2_ = 557.05; *P* = 0.0018) and third (*F*_1,1_ = 1402.52; *P* = 0.0170) breakdowns.

There was no difference in the percentage of MEAM1 and MED-Q2 females collected from either host for all three breakdown intervals assessed when whiteflies were inoculated as a single species (Table 2).

**Table 2:** Mean percentage of MEAM1 and MED-Q2 ± SE females from pepper and watermelon plants when inoculated with one species at 1^st^, 2^nd^ and 3^rd^ breakdown intervals.

Breakdown	Species	Pepper	Watermelon
1	MEAM1	53.0 ± 4.14 Aa	56.25 ± 5.20 Aa
	MED-Q2	48.33 ± 10.73 Aa	47.00 ± 7.78 Aa
2	MEAM1	64.00 ± 3.94 Aa	61.25 ± 2.50 Aa
	MED-Q2	42.75 ± 5.72 Aa	54.67 ± 1.45 Aa
3	MEAM1	66.00 ± 2.00 Aa	74.00 ± 1.22 Aa
	MED-Q2	52.50 ± 26.50 Aa	76.00 ± 1.41 Aa

Lowercase letters are used to compare means within the same column and breakdown group; uppercase letters are used to compare means in the same row. Means within the same cell (Breakdown × Species) followed by different lowercase letters are significantly different at *P* = 0.05; means within the same row followed by a different uppercase letter are significantly different at *P* = 0.05.

Regarding the life history parameters (Table 3), the mean number of eggs laid was higher for MEAM1 species than MED-Q2 species on pepper (*F*_1,9_ = 71.46; *P* < .0001) and watermelon (*F*_1,6_ = 127.73; *P* < .0001). The mean number of nymphs of MEAM1 species present on the leaves was also higher than the mean number of nymphs of MED-Q2 on pepper (*F*_1,9_ = 36.88; *P* = 0.0002) and watermelon (*F*_1,6_ = 102.34; *P* < .0001). The percentage hatch did not differ between both *B*. *tabaci* species on pepper (*F*_1,9_ = 0.37; *P* = 0.5605) and watermelon (*F*_1,5_ = 0.00; *P* = 0.9988).

**Table 3:** Mean number per leaf of eggs laid, first instar nymphs and percentage egg hatch of MEAM1 vs MED-Q2 on pepper and watermelon.

Host plant	Whitefly species	Eggs laid (±SE)	First instar nymphs (±SE)	Percentage hatch (±SE)
Pepper	MEAM1	26.00a (±4.52)	14.10a (±2.81)	52.0a (±4.0)
	MED-Q2	9.40b (±3.03)	5.30b (±1.96)	38.0a (±11.0)
Watermelon	MEAM1	39.30a (±7.55)	27.66a (±5.01)	66.0a (±9.00)
	MED-Q2	7.33b (±3.25)	3.25b (±0.88)	70.0a (±13.0)

Means followed by different letters within columns indicate significant difference (p < 0.05) between MEAM1 and MED-Q2 on each host plant for each parameter evaluated.

**Fig 2:**
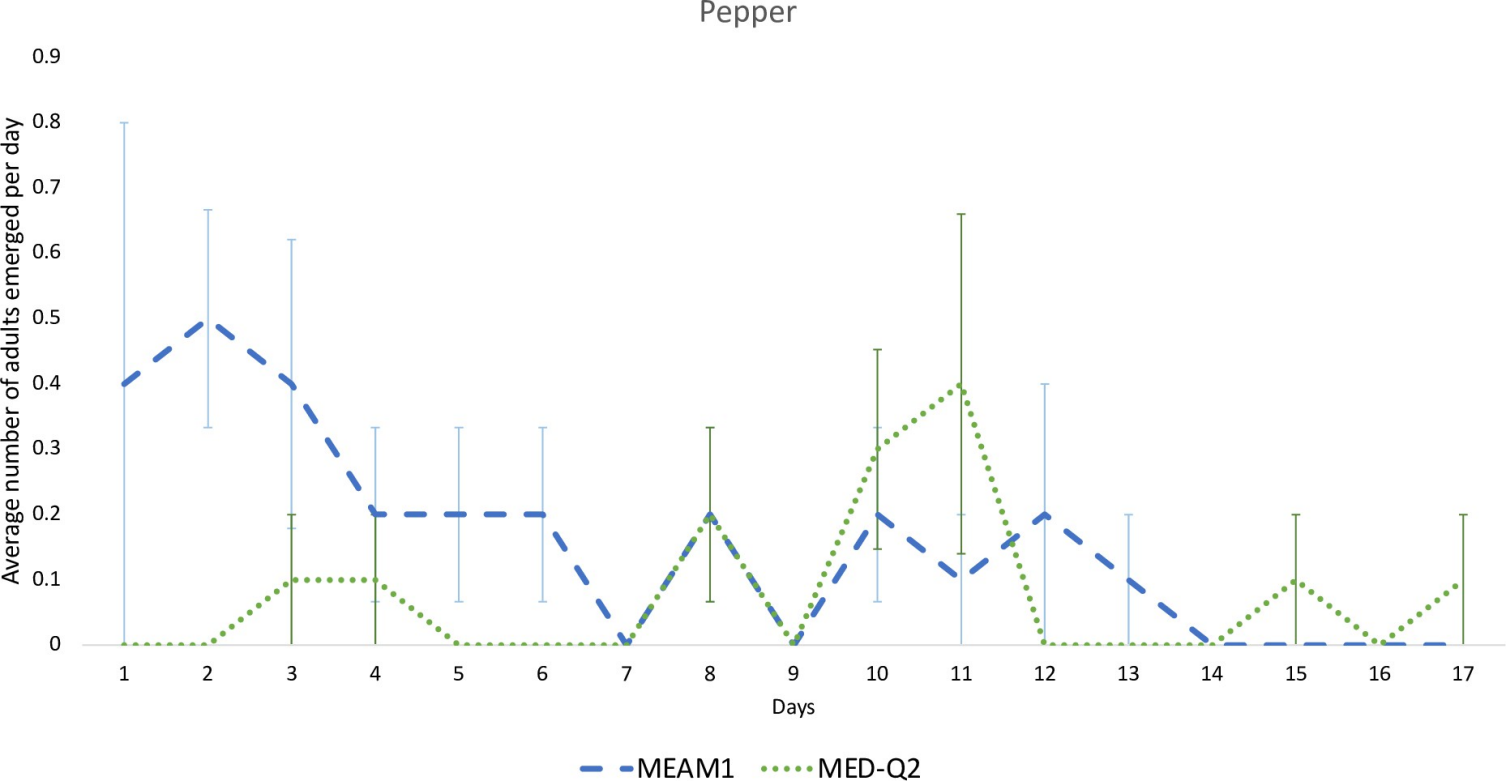
Number of adults emerged for MEAM1 and MED-Q2 per day on pepper since the first emergence recorded.

The emergence of adults on pepper is shown in Fig 2. Pepper favors the emergence of MEAM1 in the first week after the first emergence. The emergence of MED adults is higher in the second week. The earlier emergence of MEAM1 can be an advantage to outcompete MED on pepper.

The phylogenetic tree (S1 Fig) grouped populations from previous field surveys carried out in the US into two different clades, 14 populations into the MED-Q1 clade and 22 populations into the MED-Q2 clade. The MED populations from previous competition studies between MED and MEAM1 from China (GQ371165) and Brazil (KX673609) were both classified as belonging to the MED-Q1 mitochondrial group. In contrast, the MED population used in this study from Florida (GenBank Accession OK086082) was placed into the MED-Q2 mitochondrial group.

## Discussion

The competitive displacement and the life history results suggest that both pepper and watermelon are less suitable for the *B*. *tabaci* MED-Q2 mitochondrial group compared to MEAM1. In contrast, several previous studies conducted in different countries, including China [25], Japan [34] and Brazil [24], have found a higher ability of MED to colonize pepper compared to MEAM1. The phylogenetic analysis revealed that both populations, from the competition studies conducted in China and Brazil, were grouped in the MED–Q1 clade, whereas the Florida population was placed in the MED–Q2 clade (S1 Fig). These results align with a recent report showing differences in suitability on different plant hosts among the MED mitochondrial groups and suggesting that MED-Q1 is more polyphagous than MED-Q2 [21]. That study evaluated several characteristics such as oviposition, leaf preference, survival, fecundity and sex ratio for 13 different plant hosts and ranked the most suitable host accordingly. It was found that pepper was the least suitable host for a *B*. *tabaci* MED—Q2 population from Israel. In contrast, pepper was the 5^th^ and 8^th^ most suitable host for two *B*. *tabaci* MED—Q1 populations tested from Spain and Sudan, respectively. Therefore, combining the data herein obtained and the results from the above-mentioned study, we have evidence of the association between the Q2 mitochondrial group of *B*. *tabaci* MED species and low suitability on pepper.

These results may have significant implications for whitefly management and for regulation of *B*. *tabaci* MED in areas where it has not yet achieved pest status. The phylogenetic analysis (Fig 1) also revealed that both mitochondrial groups (Q1 and Q2) are present in the US indicating that other factors might be contributing to the fact that MED has not achieved pest status on pepper or other crops in the US. The primary driver behind displacement of MEAM1 by MED is insecticide use [9, 18]. However, the genetic component of *B*. *tabaci* MED must be taken in consideration as an important factor for MED reaching a pest status depending on the crop.

Studies performed in the Mediterranean region, which is the center of origin of *B*. *tabaci* MED species, have shown that a significant factor determining the predominant MED mitochondrial group is the type of cultivation. The presence of MED-Q2 was associated with greenhouse crops whereas MED-Q1 prevailed in open-field cultivation and weeds in studies from Italy [35, 36] and Greece [13]. Recently in Brazil, whitefly outbreaks in the pepper crop associated with the MED species were reported in São Paulo and Parana states in greenhouse and open field conditions [17].

In Florida, MEAM1 does not tend to develop to economically damaging levels in field produced pepper, where broad mites (*Polyphagotarsonemus latus*), thrips (Thysanoptera) and pepper weevil (*Anthonomus eugenii*) are the primary pests [37]. MEAM1 can reach high numbers on peppers produced in protected structures such as greenhouses and high tunnels in Florida. By contrast, MEAM1 is a serious pest annually of watermelon and other field grown cucurbits in Florida, including squash and cucumbers. Watermelon is infected by three whitefly transmitted viruses in Florida: Cucurbit leaf crumple virus, Cucurbit yellow stunting disorder virus and Squash vein yellowing virus [38]. Our results are consistent with field observations that watermelon tends to be a much better host for MEAM1 than pepper, although by the third breakdown date numbers of MEAM1 on pepper were consistent with densities that are sometimes observed in protected culture. While watermelon was also a better host than pepper for MED-Q2, in the sense that numbers were higher on watermelon, numbers overall were lower on each host for MED-Q2 than for MEAM1. When MED first became established on landscape plants in Florida in 2016, one of the primary concerns was that it would transmit plant viruses more efficiently than MEAM1 [7]. The risk associated with MED was based on research which in many cases involved MED-Q1 or research in which the subclade of MED was not identified. Subsequent research, including the information presented in this report, confirms that pest status varies among MED subclades. The findings from this study combined with previous research reinforce that biological and behavioural characteristics of MED species are strongly linked to intraspecific genetic variability. Although MED was detected in multiple landscape sites in 2016 in Florida, there is no evidence that it has displaced MEAM1 as has been observed in other regions [8, 11, 14, 18]. This is consistent with the fact that MED has been detected in multiple states of the United States since 2005, yet has not produced economically damaging populations on a field or regional level anywhere in the country [6, 7]. The ability of MEAM1 to outcompete other subclades of *B*. *tabaci* in the absence of insecticide selection pressure is well documented [39, 40]. This ability is related to more aggressive mating behaviour on the part of MEAM1 [39, 40]. It is essential to monitor and characterize the populations of MED according to genetic as well as behavioral factors to determine pest status as some genetic groups might be more harmful and more suitable to certain crops compared to others.

## Supporting information

S1 Fig Phylogenetic tree of the partial mtCOI gene from Mediterranean species of the *Bemisia tabaci* complex. The sequences used in previous whitefly competition studies as well as the sequences used in the current competition study are highlighted.
(TIF)
