# Supplementary figures and images for: Evidence of the association between the Q2 mitochondrial group of Bemisia tabaci MED species (Hemiptera: Aleyrodidae) and low competitive displacement capability
Source: PLoS One. 2023 Jan 12;18(1):e0280002. doi: 10.1371/journal.pone.0280002 (PMC9836299; doi:10.1371/journal.pone.0280002)

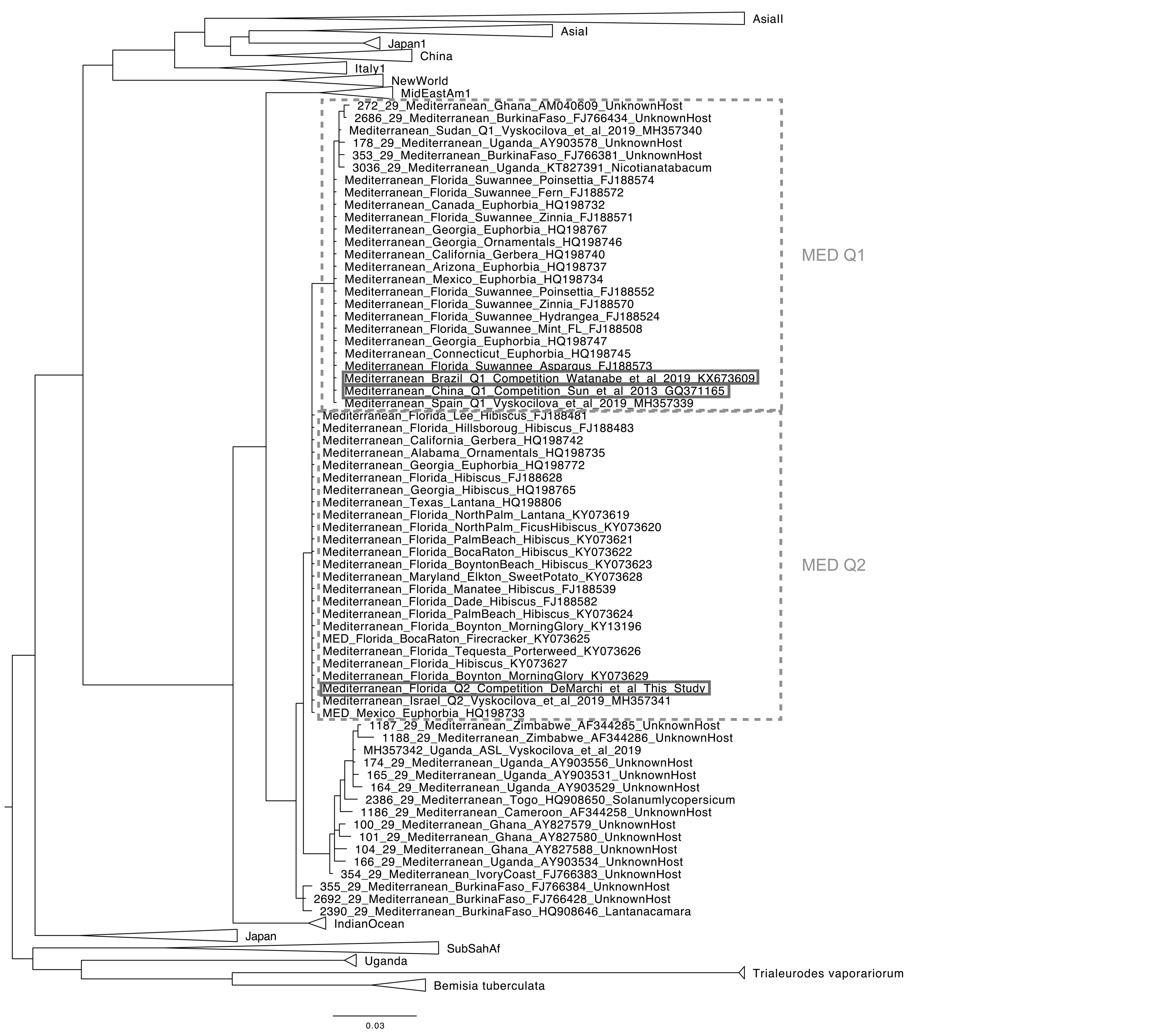

Supplement: S1 Fig — The sequences used in previous whitefly competition studies as well as the sequences used in the current competition study are highlighted. (TIF) [file pone.0280002.s001.tif]
